# Sigmoid Colon Perforation in Diffuse Large B-Cell Lymphoma Due to Tacrolimus-Induced Immunodeficiency: A Case Report

**DOI:** 10.7759/cureus.54571

**Published:** 2024-02-20

**Authors:** Shunsuke Sakuraba, Kazumasa Nakamura, Kohei Koido, Hiroyuki Hazama, Kou Ohata

**Affiliations:** 1 Gastrointestinal Surgery, Shizuoka General Hospital, Shizuoka, JPN

**Keywords:** gastrointestinal lymphoma, tacrolimus, iatrogenic immunodeficiency, sigmoid colon perforation, dlbcl

## Abstract

The sigmoid colon is an uncommon site for the origin of primary malignant lymphomas in the GI tract. Additionally, immunosuppressive agents, widely used in treating autoimmune diseases, have been associated with the induction of malignancies, including lymphoproliferative disorders. In this report, we present a rare case of GI perforation suggesting a link between immunosuppressive therapy, particularly tacrolimus treatment, and diffuse large B-cell lymphoma (DLBCL). A 75-year-old female patient presented with abdominal pain to our ER. She had a medical history of polymyositis and interstitial pneumonia, treated with the immunosuppressant tacrolimus. An abdominal CT scan revealed free gas in the abdominal cavity, leading to a diagnosis of GI perforation. The patient exhibited generalized peritonitis and underwent emergency surgery the same day. During surgery, a perforation in the sigmoid colon was identified, and a Hartmann procedure was performed. Postoperative pathology showed CD20+, CD30+, CD5-, CD10-, BCL6+, MUM1+, and MIB-1 LI of 50-60%. The diagnosis of DLBCL was confirmed, classified as EBV-positive diffuse large B-cell lymphoma, not otherwise specified (NOS), in the sigmoid colon, with positive EBER-ISH, LMP-1, and EBNA2 findings. Given her history of immunosuppressant use, she was categorized as having other iatrogenic immunodeficiency-associated lymphoproliferative disorders (OIIA-LPD), according to the WHO Classification of 2017. This case highlights the importance for clinicians to consider the risk of oncogenesis associated with the prolonged use of immunosuppressive agents.

## Introduction

Primary malignant lymphomas of the gastrointestinal tract account for 30-45% of extranodal lymphomas, making them the most common type. While they mainly present as non-Hodgkin's lymphoma, comprising 4-20% of all non-Hodgkin's lymphomas [1-3], lymphomas originating in the colon are comparatively rare [4]. Gastrointestinal perforation is a recognized complication in the treatment of gastrointestinal malignant lymphoma. However, cases where the lymphoma is first identified through postoperative pathological analysis following colorectal perforation are uncommon. Furthermore, the use of immunosuppressive drugs is known to induce lymphoproliferative diseases, classified as 'other iatrogenic immunodeficiency-associated lymphoproliferative disorders' (OIIA-LPD) according to the WHO classification. This study reports a unique case of diffuse large B-cell lymphoma (DLBCL; classified as OIIA-LPD) in the sigmoid colon, diagnosed post-emergency surgery for sigmoid colon perforation.

## Case presentation

**Figure 1:**
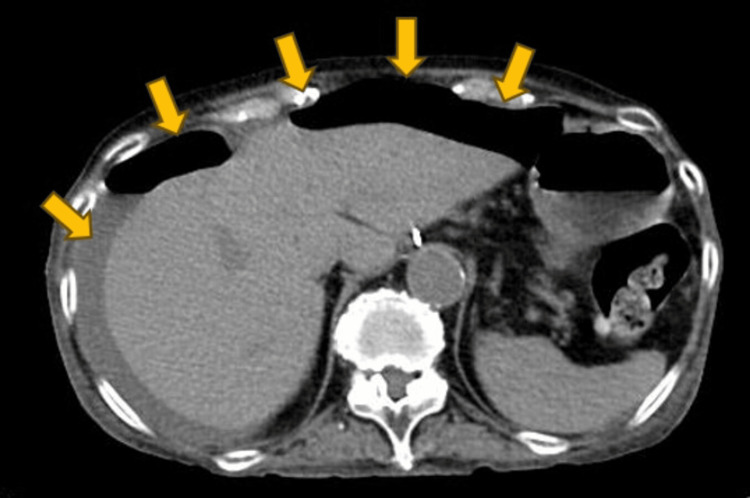
Free gas is sporadically observed in the abdominal cavity, and a moderate amount of ascites is present.

**Figure 2:**
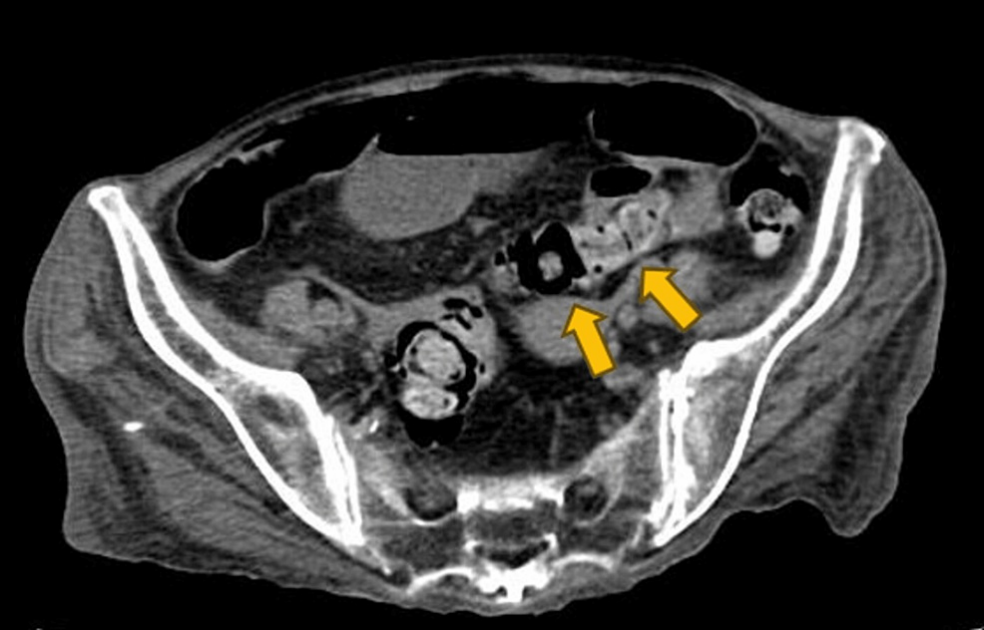
Retrospective analysis revealed no evidence of a tumor suggestive of malignant lymphoma in the sigmoid colon.

A 75-year-old woman presented to our emergency room with abdominal pain. She had a medical history of cholecystectomy for gallbladder cancer and was managing diabetes mellitus, polymyositis, and interstitial pneumonia as comorbidities. Her medications included prednisolone sodium succinate (20 mg/day), tacrolimus hydrate (6 mg/day), rabeprazole sodium, trimethoprim-sulfamethoxazole, dapagliflozin propylene glycolate hydrate, sitagliptin phosphate hydrate, and repaglinide. Upon arrival, her vital signs were: temperature 37.1°C, blood pressure 118/78 mmHg, respiratory rate 35, heart rate 115. Physical examination revealed signs of peritoneal irritation throughout the abdomen. Blood tests indicated an elevated inflammatory response (Table 1). An abdominal CT scan showed free gas in the cavity (Figure 1), indicating gastrointestinal perforation, though the specific site was unclear (Figure 2).

**Table 1:** Blood test indicated an elevated inflammatory response. Hb: Hemoglobin; Plt: Platelet; AST: Aspartate aminotransferase; ALT: Alanine aminotransferase; T. Bil: Total bilirubin; Alb: Albumin; BUN: Blood urea nitrogen; Cre: Creatinine; CRP: C-reactive protein.

Parameter	Value	Reference range
WBC	25600	3300-8600 /µL
RBC	401	386-492 ×10^4^/µL
Hb	11.6	11.6-14.8 g/dL
Plt	19	15.8-34.8 ×10^4^/µL
AST	18	13-30 U/L
ALT	15	7-23 U/L
T. Bil	0.6	0.4-1.5 mg/dL
Alb	2.5	4.1-5.1 g/dL
BUN	46	8-20 mg/dL
Cre	1.28	0.46-0.79 mg/dL
CRP	41.97	0.00-0.14 mg/dL

**Figure 3:**
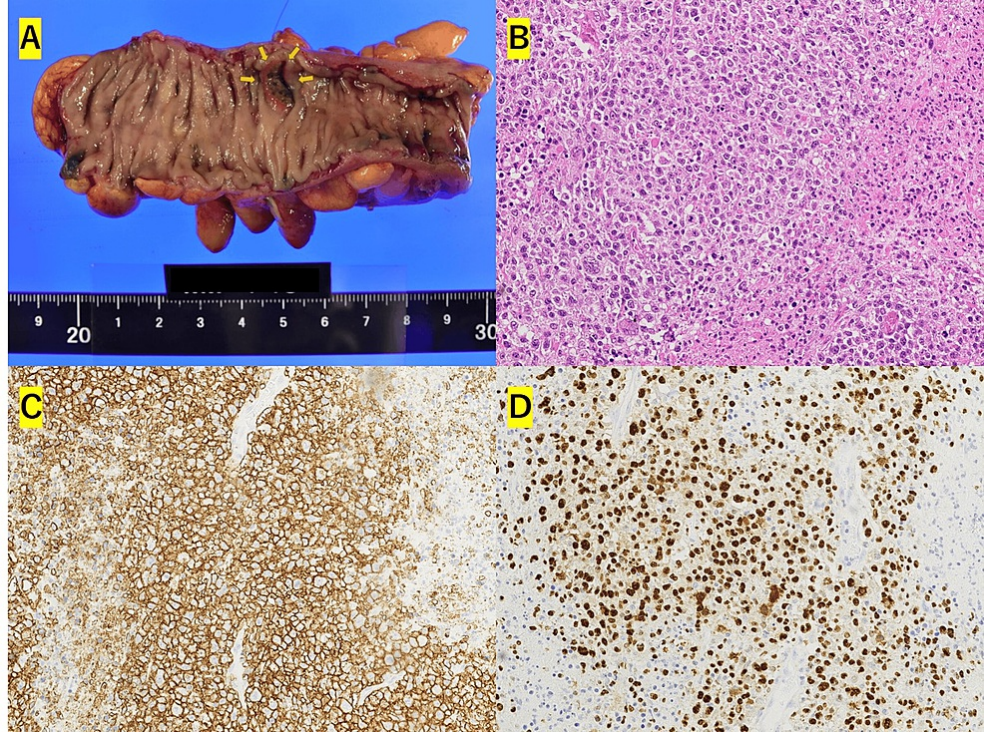
Pathological examination findings. (3A): Macroscopic findings of the excised specimen. (3B): H&E stain, magnification ×20. (3C): Immunohistochemistry showing CD20+ cells, magnification ×20. (3D): EBER in situ hybridization positive (EBER-ISH+), magnification ×20.

Emergency surgery for generalized peritonitis due to gastrointestinal tract perforation revealed a 5 mm perforation in the sigmoid colon. A Hartmann procedure was performed. The histopathological examination of the resected sigmoid colon revealed a 12 x 15 mm tumor formation consistent with the area of ulceration (Figure 3A). The tumor had infiltrated from the mucosal layer to the submucosal layer, where there was a dense proliferation of large atypical lymphocyte-like cells (Figure 3B). The interior of the tumor was necrotic and opened into the lumen. Immunohistology was positive for CD20+, CD30+, CD5-, CD10-, BCL6+, MUM1+, MIB-1 labeling index of 50-60%. EBER-ISH, LMP-1+, and EBNA2+ confirmed the diagnosis of EBV-positive DLBCL, NOS, in the sigmoid colon (Figures 3C-3D).

Based on her history of immunosuppressive drug use, she was classified as having other OIIA-LPD according to the WHO Classification of 2017. The postoperative course was uneventful, with no complications such as surgical site infections (SSIs). Postoperative CT scans showed no further lesions or enlarged lymph nodes, leading to a diagnosis of primary malignant lymphoma of the sigmoid colon. Following surgery, her general condition was graded as 3 on the ECOG Performance Status Scale, and she was transferred to a hospital for best supportive care, without undergoing additional detailed examination or treatment.

## Discussion

Approximately 1-8% of GI malignancies are considered to be primary malignant lymphomas of the GI tract [5,6]. Among these, the stomach is the most common primary site, followed by the small intestine and the large intestine [7,8]. Colorectal primary malignant lymphoma is particularly rare, accounting for only 0.16% of colorectal malignancies [9]. Within the large intestine, lymphomas most commonly originate in the cecum and rectum. The rarity of the sigmoid colon as a site for GI malignant lymphomas could be attributed to the distribution of immune tissue, which is thought to influence the site of origin [4]. According to Lewin KJ et al. (1978), primary malignant lymphoma of the GI tract is defined as a GI primary if the lesion is predominantly located in the GI tract, regardless of the disease stage [10].

Other iatrogenic immunodeficiency-associated lymphoproliferative disorders (OIIA-LPD), similar to HIV infection and post-transplantation lymphoproliferative disorders, are associated with immunodeficiency. Immunosuppressive drugs, such as methotrexate and tacrolimus, have been implicated in their causation. Cases of OIIA-LPD are often positive for Epstein-Barr virus (EBV) [11], and our case was classified as EBV-positive DLBCL, not otherwise specified (NOS), according to the immunohistological findings. This disease category, recognized in the 2017 WHO revision, was previously known as age-related EBV-associated B-cell lymphoproliferative disorder [12].

Among OIIA-LPDs induced by immunosuppressive drugs, methotrexate-associated lymphoproliferative disorders (MTX-LPD) are the most well-known [13,14]. While there are fewer reports of tacrolimus-induced malignant lymphomas compared to MTX-LPD, tacrolimus is still considered a causative agent for OIIA-LPD [15,16]. In this case, the final diagnosis was considered to be classified as OIIA-LPD because of the use of tacrolimus, an immunosuppressive drug. Recent studies suggest that topical tacrolimus therapy does not significantly increase the risk of skin cancer or malignant lymphoma [17]. Cessation of the suspected drug is a treatment option for MTX-LPD, with some cases achieving remission following drug discontinuation, typically showing tumor regression within two weeks [18,19]. Patients who respond to drug cessation should be closely monitored, and if remission is not achieved, treatment for malignant lymphoma should be considered. The evidence for drug cessation in tacrolimus-induced malignant lymphoma is less established than for MTX-LPD, but cases of remission following tacrolimus discontinuation have been reported. Drug cessation could thus be considered a viable treatment option in such cases [16]. In our case, discontinuation of tacrolimus was contemplated as a future treatment strategy, though the final decision was deferred to the physician managing the patient's dermatomyositis and interstitial pneumonia.

Due to the rarity of cases, coherent data on the prognosis of colorectal lymphoma are limited. However, a case report summarizing 42 patients with colon lymphoma reported an overall survival of 77 months (range: 25-180 months) [20]. Other scattered reports suggest a generally poor prognosis for this disease [9]. Given the diverse classifications and histologic types of malignant lymphomas, and the paucity of reports on lymphomas originating in the colon, it is challenging to generalize their prognoses.

## Conclusions

In this case, the use of the immunosuppressive drug tacrolimus appears to have played an important role in the development of lymphoma. Our findings emphasize the need for clinicians to consider the potential risk of oncogenesis associated with the prolonged administration of immunosuppressive medications.
